# Transcriptome analysis of microglia in a mouse model of Rett syndrome: differential expression of genes associated with microglia/macrophage activation and cellular stress

**DOI:** 10.1186/s13229-017-0134-z

**Published:** 2017-03-29

**Authors:** Dejian Zhao, Ryan Mokhtari, Erika Pedrosa, Rayna Birnbaum, Deyou Zheng, Herbert M. Lachman

**Affiliations:** 1 Department of Genetics, Albert Einstein College of Medicine, 1300 Morris Park Ave., Bronx, NY USA; 2 Department of Psychiatry and Behavioral Sciences, Albert Einstein College of Medicine, 1300 Morris Park Ave., Bronx, NY USA; 3 Department of Neurology, Albert Einstein College of Medicine, 1300 Morris Park Ave., Bronx, NY USA; 4 Department of Neuroscience, Albert Einstein College of Medicine, 1300 Morris Park Ave., Bronx, NY USA; 5 Department of Medicine, Albert Einstein College of Medicine, 1300 Morris Park Ave., Bronx, NY USA

**Keywords:** Microglia, Rett syndrome, Autism, Schizophrenia, Heat shock, M1 activation, M2 activation, Innate immune system

## Abstract

**Background:**

Rett syndrome (RTT) is a severe, neurodevelopmental disorder primarily affecting girls, characterized by progressive loss of cognitive, social, and motor skills after a relatively brief period of typical development. It is usually due to de novo loss of function mutations in the X-linked gene, *MeCP2*, which codes for the gene expression and chromatin regulator, methyl-CpG binding protein 2. Although the behavioral phenotype appears to be primarily due to neuronal Mecp2 deficiency in mice, other cell types, including astrocytes and oligodendrocytes, also appear to contribute to some aspects of the RTT phenotype. In addition, microglia may also play a role. However, the effect of *Mecp2* deficiency in microglia on RTT pathogenesis is controversial.

**Methods:**

In the current study, we applied whole transcriptome analysis using RNA-seq to gain insight into molecular pathways in microglia that might be dysregulated during the transition, in female mice heterozygous for an *Mecp2*-null allele (*Mecp2* ^+/−^; Het), from the pre-phenotypic (5 weeks) to the phenotypic phases (24 weeks).

**Results:**

We found a significant overlap in differentially expressed genes (DEGs) with genes involved in regulating the extracellular matrix, and those that are activated or inhibited when macrophages and microglia are stimulated towards the M1 and M2 activation states. However, the M1- and M2-associated genes were different in the 5- and 24-week samples. In addition, a substantial decrease in the expression of nine members of the heat shock protein (HSP) family was found in the 5-week samples, but not at 24 weeks.

**Conclusions:**

These findings suggest that microglia from pre-phenotypic and phenotypic female mice are activated in a manner different from controls and that pre-phenotypic female mice may have alterations in their capacity to response to heat stress and other stressors that function through the HSP pathway.

**Electronic supplementary material:**

The online version of this article (doi:10.1186/s13229-017-0134-z) contains supplementary material, which is available to authorized users.

## Background

Rett syndrome (RTT) is characterized by a progressive loss of cognitive, social, and motor skills after a relatively brief period of typical development. It is found in ~1/10,000–1/15,000 female births and is usually due to de novo loss of function mutations in the X-linked gene, *MeCP2*, which codes for methyl-CpG binding protein 2 (MeCP2) [1]. MeCP2 binds to methylated cytosine at CpG islands, which are the canonical DNA methylation sites, as well as to non-CpG cytosine residues, and 5-hydroxymethylcytosine [2–4]. It has also recently been found to inhibit microRNA processing [5]. Although severe loss of function mutations are usually male-lethal, hypomorphic *MeCP2* variants have been found in males with intellectual disability and behavioral deficits [6, 7].

Rarely, mutations in *CDKL5*, *SHANK3*, *FOXG1*, *ANKRD31*, and *CHRNA5* cause a RTT-like phenotype [8–10].

One of the more perplexing aspects of RTT is the loss of previously acquired developmental milestones, which occurs after ~6–18 months of age. Regression is characterized by loss of language skills, reduced brain growth, repetitive stereotyped hand movements, and impaired motor skills [1, 2]. Following this period of regression, the clinical picture stabilizes for a while, but ultimately, motor deterioration, autistic features, seizures, growth failure, autonomic dysfunction, and gastrointestinal disturbances emerge.

In addition to RTT, de novo mutations in *MeCP2* can contribute to schizophrenia (SZ) risk in a small subgroup of individuals [11]. And a recent genome wide association study (GWAS) carried out in a Han Chinese cohort suggests that common variants in *MeCP2* might also play a role in this condition [12].

Although Mecp2 is ubiquitously expressed, most studies point to neuronal dysfunction as a primary cause. For example, a number of different neuron-specific *Mecp2* KO mice show functional abnormalities [13–15]. In addition, restoring Mecp2 expression in neurons normalizes brain weight and activity and extends lifespan [16]. An increase in cell packing density and a reduction in the complexity of neuronal dendritic branching have also been found, as well as alterations in dendritic spine numbers and synaptic architecture [17, 18]. Selective loss of *Mecp2* in gamma-aminobutyric acid-ergic (GABAergic) inhibitory interneurons recapitulates most of the RTT phenotype [19]. However, some RTT features are also seen when selective loss of expression is induced in excitatory glutamatergic neurons [20]. In addition, the absence of *Mecp2* has been found to cause a decrease in excitatory synapses and to impair long-term potentiation, an effect that was found in severely phenotypic, but not pre-phenotypic mice [21]. Interestingly, *MeCP2* duplications also cause neurodevelopmental problems in humans and mice and are associated with increased synaptogenesis and dendritic complexity in vitro [2, 22, 23].

Although a primary neuronal dysfunction is clearly important in RTT, neurogenesis and synaptic function are modulated by other cell types in the brain—astrocytes and microglia, for example—and loss of function abnormalities in *MECP2* in these cells could conceivably play a role in some aspects of the RTT phenotype. This idea is supported by several studies. For example, reduced *Mecp2* expression in astrocytes negatively influences neuronal function, and reexpression improves locomotion and anxiety symptoms, restores respiratory abnormalities and dendritic morphology, and increases lifespan [24]. In addition, *Mecp2*-deficient astrocytes and conditioned medium from these cells failed to support normal dendritic morphology of wild-type (WT) and *Mecp2*-deficient hippocampal neurons. Also, mice with oligodendrocyte lineage-specific reduction of *Mecp2* develop severe hindlimb clasping phenotypes, and restoration in an RTT model improved locomotor deficits and hindlimb clasping in both male and female mice, and restored body weight in males [25].

Finally, microglia have been implicated in RTT. Jin et al., for example, found that an increase in the expression of *Slc38a1* (which codes for a glutamine transporter) caused by *Mecp2* deficiency results in a glutamine-dependent decrease in microglia viability and a reduction in brain microglial number through the production of reactive oxygen species (ROS), which causes mitochondrial dysfunction and neurotoxicity [26]. In addition, a defect in microglial phagocytosis, which is a key homeostatic process in maintaining normal synaptic architecture, has been found in an RTT model [27]. Remarkably, phagocytic activity restored by a peripheral bone marrow transplant from WT mice, which led to the engraftment of phagocytic cells in the brain, rescued many aspects of the RTT phenotype in a mouse model; an increase in body weight and locomotor activity occurred, and breathing patterns were normalized [27]. Wang et al., however, were unable to replicate this finding, casting some doubt on the role of microglia in RTT [28]. In addition, Schafer et al. recently found that while microglia may contribute to the RTT phenotype by enhancing engulfment and elimination of presynaptic inputs at the end stages of disease, specific loss or gain of *Mecp2* expression in microglia were not primary event [29].

To further address the potential role of microglia in RTT, we now report a transcriptome analysis in female Het mice. Transcriptomes were assessed during the transition from the RTT pre-phenotypic to the phenotypic stages (5 and 24 weeks, respectively). This time course models the natural progression of clinical RTT. In addition, our study is relevant in that, we used female mice; studies with mouse models of RTT rarely use females because symptoms occur earlier and are more severe in males, yet females account for nearly all RTT cases.

## Methods

### Rett mouse model

The B6.129P2(C)-*Mecp2* ^*tm1.1Bird*^/J mouse model developed in the Adrian Bird lab [30] was used for these experiments. They were obtained from The Jackson Laboratory (Bar Harbor, Maine; stock number 003890). C57BL/6 mice were used as controls (stock no: 000664). The protocol for generating the KO strain is described at https://www.jax.org/strain/003890 and in the original paper [30]. Briefly, a targeting vector was designed to insert loxP sites around *Mecp2* exons 3 and 4. The construct was transfected into 129P2/OlaHsd-derived E14TG2a embryonic stem (ES) cells. The engineered ES cells were injected into C57BL/6 blastocysts. Chimeric offspring were bred to C57BL/6 mice to produce heterozygous floxed females. The floxed line was bred to homozygosity. Homozygous floxed female mice were crossed with male CMV-Cre mice to generate heterozygous Het females.

The mice were housed at the Albert Einstein College of Medicine in our central AAALAC-accredited animal facility on a 14-h day and 10 night light cycle, and were fed a regular diet. All animals were sacrificed in the morning within a 1-h window to avoid potential circadian differences in gene expression. Heterozygosity for the KO allele was confirmed by PCR using primers 9875 (aaattgggttacaccgctga); 9877 (ccacctagcctgcctgtact); and oimr7172 (ctgtatccttgggtcaagctg), according to the protocol recommended by The Jackson Laboratory (https://www2.jax.org/protocolsdb/f?p=116:5:0::NO:5:P5_MASTER_PROTOCOL_ID,P5_JRS_CODE:8898,003890). Three RTT mice and three controls were used at each time point (total of 12).

The 24-week Het female mice displayed a classic hindlimb clasping phenotype, a response indicative of general neurological pathology that is observed in the mouse model of RTT [25, 31]. Mice were scored on 3 consecutive days after beginning to show the reflex between weeks 23–24. They were rated based on severity of hindlimb clasping on a 0–3 scale [31]. The mice were suspended by the base of their tail and video recorded for 10–15 s. At 24 weeks, all mice scored 3 (most severe) with hindlimbs completely retracted inwards towards the abdomen for more than 50% of the recorded time.

### Microglial isolation

Microglia were isolated from whole brains using the Miltenyi MACS® (Magnetic Cell Isolation and Cell Separation) protocol, which contains the Neural Dissociation kit (P), Mylein Removal Beads II, and CD11b antibody bound to Microbeads, along with the gentleMACS Dissociator, according to the manufacturer’s protocol. Briefly, the entire brain was isolated and the meninges were removed by manual dissection. The brain was roughly chopped and subsequently processed using the Neural Tissue Dissociation kit (P) protocol for the gentleMACS Dissociator (programs m_brain_01_02, m_brain_02_02, and m_brain_03_01). Samples were then further processed for myelin removal and the isolation of CD11b-positive cells. Microglial isolation was confirmed by FACS with a monoclonal CD11b antibody conjugated to PE (Miltenyi). The microglial isolation protocol typically results in a population of cells that contain ~80% microglia (Additional file 1: Figure S1).

### RNA extraction

Total RNA was extracted using a miRNeasy Kit according to the manufacturer’s instructions (Qiagen). An additional treatment with DNase1 (Qiagen, Valencia, CA) was included to remove genomic DNA. Reverse transcribed PCR (RT-PCR) was performed using a OneStep RT-PCR Kit (Qiagen, Valencia, CA) according to the manufacturer’s instructions.

### RNA-seq

After passing quality control, high-throughput sequencing libraries were prepared by the Einstein Epigenomics and Genomics Shared Facility following the standard protocol established for RNA-seq on the Illumina platform. We obtained 101 bp paired-end RNA-seq reads from an Illumina HiSeq 2500 instrument. Adapters and low-quality bases in reads were trimmed by trim_galore (http://www.bioinformatics.babraham.ac.uk/projects/trim_galore/). RNA-seq reads were aligned to the mouse reference genome (GRCm38/mm10) using Tophat (v2.0.13) in a strand-specific manner (--library-type fr-firststrand) [32]. Uniquely, mapped reads were counted for each gene using “htseq-count” in HTSeq package (v0.6.1) with gene models from Ensembl release 75 (--stranded=reverse --minaqual=10 --type=exon --idattr=gene_id --mode=union) [33]. DESeq2 was used to identify differentially expressed genes (DEGs) (adjusted *p* value <0.05) [34]. The ToppGene suite was used with default settings to identify functional enrichment in the DEG lists (http://toppgene.cchmc.org) [35]. ToppGene assesses gene lists for functional enrichment based on transcriptome studies, gene ontologies (GO, pathway), human diseases and mouse phenotypes, and literature citation.

### Microarray analysis

Our DEG lists were compared with genes activated in mouse macrophage during the transition from the M0 to the M1 and M2 activation states. The normalized expression values were downloaded from the GEO database (GSE69607), which was generated by the original authors with the RMA algorithm [36]. As the authors did not provide their lists of DEGs, we applied the limma package ver. 3.26.8 to detect DEGs [37]. Using fold change >2 and adjusted *p* values <0.05 and the BH method [38], we obtained 1780 genes that changed during M1 or M2 activation. Of the 146 down-regulated and 82 up-regulated genes that were shared between the M1 and M2 activation states provided in the original paper [36], 113 and 74 were also identified as differentially expressed in our reanalysis, respectively, indicating that our analysis obtained similar results.

### Quantitative real-time PCR (qPCR)

Quantitative real-time PCR (qPCR) was carried out as previously described using the 2^−∆∆Ct^ method to calculate relative expression levels, with β2-microglobulin (*β2M*) and *G6PD* as reference genes [39, 40]. Briefly, cDNA was generated using iScript cDNA Synthesis Kit (Bio-Rad). qPCR was carried out using the ABI 7900HT Real-Time PCR System (Applied Biosystems, Foster City, CA). Each reaction consisted of cDNA, primers, and Power SYBR Green PCR Master Mix (Applied Biosystems, Foster City, CA) in an 8 μl volume. Melting curve analysis of target sequences showed that all primers used in this study generated amplicons that had a single peak, without primer-dimer artifacts. Primer concentrations were optimized prior to use in qPCR experiments. Each qPCR was carried out in duplicate, with each data point repeated three times. The primers used for qPCR included (forward and reverse, respectively): *G6PD* atcatgggtgcatcggtgag and agcacctgtgatggtccaag; *B2M* tgccaaaccctctgtacttc and gctaagcattgggcacagtg; *Hspa1b* tggtgctgacgaagatgaag and ccgctgagagtcgttgaagt; Hspa8-tggagaaagtctgcaacccta and tgaagaagcaccaccagatg; *Hspa1a* gctcgaatcctatgccttca and atgacctcctggcacttgtc; *Mecp2* gtccacccttggtgagaaaa and ccttcttaggtggggaggag; *Hsph1* cacgctgggatcagaatctt and acaaccacagccacacacat; *Dnaja1* cggagaggaaaactgactgc and tgcccccttagttgacaatc; cd180 cccaacagagaagctgaagg and ggctcagattagtggcttgc. Significant differences in gene expression were assessed using a two-tailed Student’s *t* test.

## Results

**Fig. 1:**
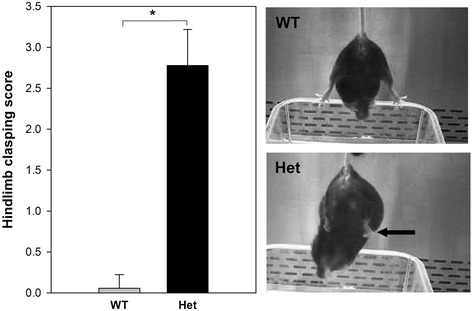
*Left panel*. Hindlimb clasping score. Het female mice were tested biweekly for the hindlimb clasping reflex and scored on a scale of 0–3 as described in the Methods section. All mice began to show the reflex after week 23. They were then scored over 3 consecutive days, and the results for each mouse were pooled. There was a significant increase in the score in the Het mice (denoted by *asterisk*, 7.4E−08, Student’s *t* test).* Right upper panel* WT mice consistently showed a lack of hindlimb clasping (outward hindlimbs) while the Het mice showed an abnormal response; clasping towards the abdomen (*arrow* *Right lower panel*)

Microglia were isolated from 5-week-old Het female mice who did not exhibit overt neurological signs and 24-week Het female mice who exhibited a significantly increased hindlimb clasping reflex (*p* = 7.4E−08, Fig. 1). Microglia were also isolated at the same time points from age-matched female WT controls. Cells were immediately frozen at −80 °C. RNA was subsequently extracted and sequenced by a strand-specific protocol (RNA-seq). The 5-week samples (Het and WT) were sequenced together in a single lane by multiplexing. The 24-week samples were similarly handled. RNA-seq reads ranged from 49,135,314–98,691,394 in the week 5 samples to 25,463,864–31,288,101 in the week 24 samples (Additional file 2: Table S1). As seen in the file, the number of mapped reads exceeded 90% for all samples and all samples passed similar quality controls.

**Fig. 2:**
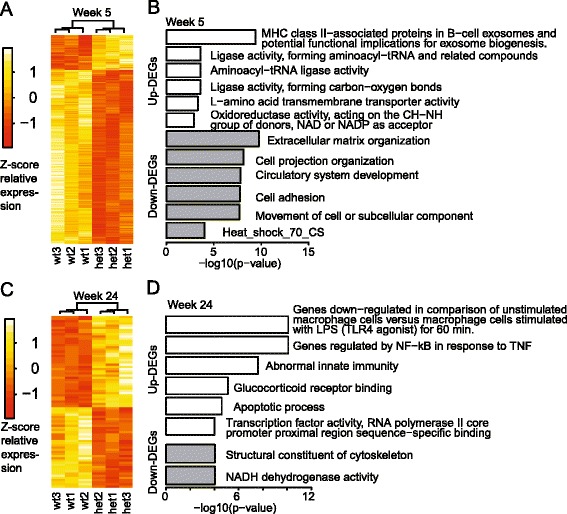
Heat map and summary of GO terms and pathways. **a**, **c** Heat maps showing differentially expressed genes in microglia between Het and WT at 5 and 24 weeks. **b**, **d** Enriched GO terms and pathways determined by the software ToppGene. The terms shown are the top enrichment terms for various categories in the DEG lists. These included gene ontology (GO) (molecular function [MF] and biological process [BP]), mouse phenotype, protein domain, co-expression, and PubMed references. All enriched terms are included in Additional file 6: Table S3

DEGs comparing WT and Het samples were identified using the DESeq2 software package and adjusted *p* values (padj) <0.05. A total of 464 DEGs were identified at 5 weeks; 78 increased in the Het samples, while 386 decreased (Additional file 3: Table S2). This was somewhat unexpected since Mecp2 is generally viewed as a transcriptional repressor, although it certainly can act as a transcriptional activator as well [41]. For the 24-week samples, 79 DEGs were found at the padj <0.05 level (42 increased in the Het samples, 37 decreased). The DEGs separated the WT (*Mecp2* ^+/+^) and Het samples (*Mecp2* ^+/−^) into two groups, as seen in the heat map (Fig. 2). In addition, Principle component analysis (PCA) using the top 5000 most variable genes, as defined by DESeq2 showed the samples could be separated, although one of the week 24 samples (Het2) showed increased variation from the other two biological replicates (Additional file 4: Figure S2).

It should be noted that the greater number of DEGs in the 5-week than 24-week samples is unlikely due to deeper sequencing depth since a similar number of week 5 DEGs were obtained using half of the reads for analysis. After randomly sampling reads from the 5-week samples five times to match the read depth of the week 24 samples, we identified between 27 and 33 up-regulated and between 224 and 257 down-regulated genes in the down-sized 5-week samples, with the same statistical thresholds (Additional file 3: Table S2). Although these numbers are smaller than those obtained with the full data set, they are still much larger than the total number of week 24 DGEs (*n* = 79), strongly suggesting that read depth is not a factor causing few DEGs in week 24 samples. Additionally, we examined the expression level of all week 5 DEGs in the week 24 samples and found that these genes exhibited similar levels of expression in the week 4 and week 24 samples (Additional file 5: Figure S3).

Among the top DEGs in the 24-week samples were *Mecp2* (padj = 0.0001). In the 5-week samples, the decrease of *Mecp2* mRNA was nominally significant (*p* = 0.002), with the adjusted *p* value falling just short of significance (0.058) (Additional file 3: Table S2).

In the 5-week samples, 24 of the top 25 DEGs were down-regulated in the Het samples. The top five were *Sema3b*, *Nov*, *Dnaja1*, *Vcam1*, and *Mgp*, three of which (*Sema3b*, *Nov*, and *Vcam1*) code for cell-cell adhesion proteins or extracellular matrix (ECM) components. This is supported by the gene list enrichment analysis using the ToppGene suite [35], which reported a significant enrichment of genes associated with the ECM among down-regulated genes, resulting in ECM being the top Biological Process GO term and cell adhesion being the third (Fig. 2; see Additional file 6: Table S3 for complete enrichment analysis).

**Fig. 3:**
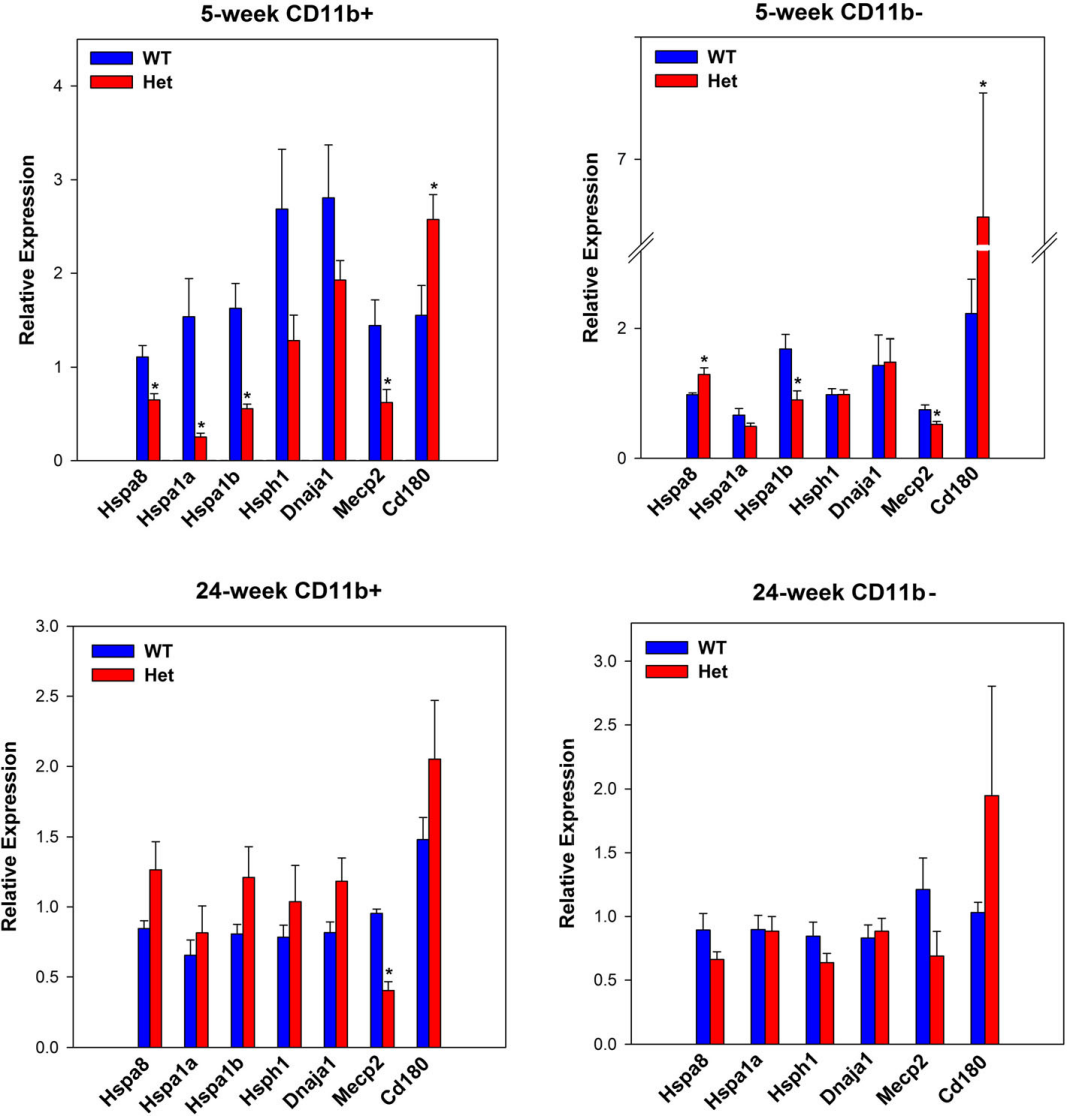
Validation of selected DEGs by qPCR. Samples were analyzed in duplicate using the 2^−∆∆Ct^ method as described in the Methods section. Significant differences between WT and Het are denoted by an asterisk (*). *p* values were derived by Student’s t test. The values are as follows: 5-week CD11b-positive fraction (microglia); Hspa8, 0.01; Hspa1a, 0.02; Hspa1b, 0.005; Hsph1, 0.08; Dnaja1, 0.2; Mecp2, 0.03, CD180, 0.03: 5-week CD11b-negative fraction; Hspa8, 0.03; Hspa1a, 0.2; Hspa1b, 0.01; Hsph1, 0.9; Dnaja1, 0.9; Mecp2, 0.01, CD180, 0.006: 24-week CD11b-positive fraction: Hspa8, 0.09; Hspa1a, 0.5; Hspa1b, 0.1; Hsph1, 0.4; Dnaja1, 0.09; Mecp2, 0.0001, CD180, 0.2: 24-week CD11b negative fraction; Hspa8, 0.1; Hspa1a, 0.9; Hspa1b, not done; Hsph1, 0.1; Dnaja1, 0.7; Mecp2, 0.1; CD180, 0.3

*Dnaja1* codes for a member of the heat shock protein (HSP) family of co-chaperones and is one of nine HSP-related genes that were significantly decreased in the 5-week Rett samples (Table 1). These findings were validated by qPCR (Fig. 3). According to the ToppGene enrichment analysis, genes coding for HSPs were the top protein domain detected among the down-regulated DEGs (Additional file 6: Table S3). In addition, qPCR analysis carried out on the CD11b-negative (non-microglia) fraction showed that at 5 weeks, *Hspa1b* was only one of the 5 HSP genes assayed that significantly decreased and a significant increase in *HSPA8* gene expression was detected. By contrast, HSP genes were not differentially expressed in the 24-week samples in either the microglia or non-microglia fractions (Fig. 3; Additional file 6: Table S3).

**Table 1:** Differentially expressed heat shock genes in 5-week RTT microglia

	5 weeks			24 weeks		
Gene	FC	*p* value	padj	FC	*p* value	padj
Dnaja1	−0.88	7.20E−14	3.78E−10	−0.02	8.58E−01	9.92E−01
Hspa8	−0.58	1.48E−11	2.59E−08	0.08	5.66E−01	9.45E−01
Dnajb1	−0.70	2.01E−11	2.88E−08	0.02	8.88E−01	9.93E−01
Hspb8	−0.96	5.09E−09	2.67E−06	0.08	5.66E−01	9.45E−01
Hsph1	−0.90	1.64E−06	2.90E−04	0.00	NA	NA
Hspa1b	−0.73	1.80E−05	2.08E−03	−0.02	7.89E−01	9.86E−01
Hspa2	−0.54	3.92E−05	3.89E−03	0.15	2.67E−01	8.50E−01
Hspa12b	−0.67	4.09E−05	3.98E−03	−0.14	3.37E−01	8.78E−01
Hsp90aa1	−0.43	4.30E−04	2.27E−02	−0.12	3.00E−01	8.59E−01

FC is log2(fold change of RTT/WT); *p* value indicates nominally significant differences, padj is the *p* value adjusted for multiple testing using the Benjamini-Hochberg method [37].

The top up-regulated genes in the 5-week samples were *C130026I21Rik*, *Cbln3*, *Gm10800*, *Retnla*, and *Rnf17*. Of these, *Retnla* is the most interesting because it is a marker of M2 macrophage/microglial activation [42]. The M1 and M2 activation states regulate pro-inflammatory and anti-inflammatory balance, respectively, in the brain and immune system, dysregulation of which has been implicated in ASD in patients and animal models (see below) [42–45].

There were fewer DEGs that showed an increase in expression in the 5-week samples, so the pathway analysis was less revealing. The top molecular function GO term was aminoacyl-tRNA ligase activity, which included the genes *Iars*, *Yars*, and *Mars*, one of which (*Yars*) has been implicated in developmental delay [46] (Additional file 3: Table S2). MHC class II was the top term based on the ToppGene analysis of PubMed literature, consistent with an effect on innate immunity (discussed below).

In the 24-week samples, the top protein-coding DEGs (up- or down-regulated) were *Cass4*, *Ifit2*, *Nfkbid*, *Rian*, and *Lsp1* (Additional file 3: Table S2). *Ifit2* codes for an interferon-inducible gene that inhibits virus replication [47]. *Cass4* codes for a scaffolding protein that has been implicated in Alzheimer’s disease, possibly through the toxic effects of Tau [48, 49]. *Nfkbid* codes for an NF-κB inhibitor [50]. Interestingly, the blood level of *NFKBID* RNA has been proposed as a biomarker in the differential response children with ASD to have atypical antipsychotic medications [51]. *RIAN* (*MEG8* in humans) is a non-coding RNA that maps to the human imprinted locus on 14q32. And *Lsp1* (lymphocyte-specific protein 1) codes for an intracellular F-actin binding protein that regulates neutrophil chemotaxis [52]. It is one of the few DEGs found in both the 5- and 24-week samples (increased expression in the Het samples for both). In addition to *Lsp1*, *Lpl* (lipoprotein lipase) was also differentially expressed at both 5 and 24 weeks. However, although *Lsp1* expression is lower in the Het samples at weeks 5 and 24, *Lpl* expression is higher in the Het samples at 5 weeks but lower at 24 weeks. Interestingly, reducing Lpl expression in BV2 microglia and primary microglial cells reduces microglial phagocytosis of fibrillar β-amyloid [53]. Correspondingly, reduced Lpl immunoreactivity has been found in granule cells of the dentate gyrus and the associated synaptic network in Alzheimer’s disease postmortem samples compared to control tissue [54]. The altered expression of *Lpl* in both sets of samples, and its differential expression between the 5- and 24-weeks samples, supports the idea that microglial phagocytosis could be disrupted in the RTT, as suggested in other studies [27, 55].

Because of the small number of DEGs, the ToppGene enrichment analysis for the 24-week DEGs should be viewed with caution. Nevertheless, several GO terms stand out. Notably, the top molecular function GO term enriched for DEGs that increased in the Het samples was glucocorticoid receptor binding, and abnormal innate immunity was the top mouse phenotype (Additional file 6: Table S3). This is consistent with a recent study showing that *Mecp2* deficiency leads to dysregulation of inflammatory responses in microglia and macrophages and to abnormalities in genes induced by glucocorticoids [56].

Similarly, the top terms for co-expression genes enriched with DEGs were related to aspects of innate immunity, including genes down-regulated in macrophage cells stimulated with LPS, a TLR4 agonist, and genes regulated by NF-kB in response to TNF.

### Comparison of DEGs with M1 and M2 activation states

Based on the finding that *Retnla* was a top up-regulated DEG in the 5-week samples and the enrichment of genes involved in innate immunity in the 24-week up-regulated DEGs, we examined our DEG lists systematically for markers of M1 and M2 activation.

For this assessment, we examined transcriptomes obtained from mouse macrophages that were stimulated towards both M1 and M2 pathways (GEO accession number GSE90736) [36]. We reanalyzed the microarray data and obtained 1780 DEGs that changed expression in activated M1 or M2 macrophages. A significant degree of overlap was found for genes associated with both the M1 and M2 activation states among the 5- and 24-week DEGs (Table 2; Additional file 7: Table S4). The smaller number of M1- and M2-activated genes among the 24-week DEGs compared to week 5 reflects the lower number of DEGs found at 24 weeks, as noted above. This does not appear to be due to differences in the depth of sequencing. We analyzed the 76 M1/M2 genes that were only differentially expressed in week 5 and found that the normalized read counts for these genes were very similar between week 5 and week 24 WT samples (Additional file 7: Table S4). This indicates that the lack of expression changes in week 24 between the Het and WT samples is unlikely affected by the lower sequencing depth.

**Table 2:** Comparison of DEGs with genes activated in M1 and M2 macrophages

Mecp2 DEGs	M1/M2 expression	Shared genes	*p* value
5 week (*n* = 464)	M1 down	28	1.83E−04
	M1 up	28	1.34E−05
	M2 down	13	3.21E−02
	M2 up	34	1.86E−12
24 week (*n* = 79)	M1 down	8	2.10E−03
	M1 up	6	1.44E−02
	M2 down	7	2.68E−04
	M2 up	8	7.90E−05

DEGs in 5- and 24-week microglia identified in this study were compared with genes activated to produce M1 and M2 macrophages from a baseline state (M0) in mouse [55]. The *p* values for the gene overlaps were determined by Fisher’s exact test

It should be pointed out, however, that despite the significant overlap, there was no correlation with the direction of change in our samples (e.g., many genes that were up-regulated in RTT microglia were down-regulated in M1 and M2 macrophages). This suggests that the M1 and M2 activation states in RTT microglia may be disrupted by Mecp2 deficiency in both pre-phenotypic and phenotypic female mice. The findings, however, do not address whether altered expression of M1- and M2-associated genes is a primary event or secondary to neuronal dysfunction that is triggering microglial activation.

These findings are again consistent with some of the findings described by Cronk et al., who showed that *Mecp2* deficiency leads to dysregulation of inflammatory responses in microglia and macrophage [56].

## Discussion

The role of microglia in ASD in general and RTT in particular is an intriguing pathophysiological perspective to consider. Several transcriptome studies show that microglia- and immune/inflammatory-associated genes are among the top DEGs in comparisons made between the ASD and control brains [45, 57, 58]. However, a key question is whether this activation is a primary or secondary phenomenon. The existing evidence points to the latter. In a study by Gupta et al., for example, a negative correlation was found between the differentially expressed neuronal module and the M2 activation state [45]. In addition, Voineagu et al. showed that the transcriptome changes they observed in the ASD brains converge with GWAS data, which are enriched for neuronal genes, in particular genes coding for synaptic proteins; immune changes have a less pronounced genetic component, suggesting that the differential expression found in ASD is secondary [58]. This idea is consistent with the key role of microglia in synaptic pruning [59–61]. A reasonable hypothesis is that a primary defect in neurons leading to dysfunctional synaptogenesis is triggering microglial activation and synaptic pruning, which is then reflected in the postmortem gene expression findings. The importance of dysfunctional synaptic pruning in neuropsychiatric disorders was highlighted in a recent study showing that allelic variation in complement component 4 (C4) genes is associated with schizophrenia: C4 is a key regulator of microglia-mediated synaptic pruning [62]. In addition, genetic studies show that genetic variation in *CSMD1*, which codes for a complement regulator, is a risk factor for both ASD and SZ [63, 64].

On the other hand, there is some support for the idea that a primary defect in microglial function could also be involved in some ASD cases. For example, disruption of genes that regulate microglial phagocytosis, such as annexin 1 (*ANXA1*) and MER proto-oncogene tyrosine kinase (*MERTK*), which are expressed at markedly elevated levels in microglia compared with neurons [65], have been found in rare, ASD-related CNVs and loss of function mutations in exome sequencing studies [66–68].

The possibility for a direct role of microglia in RTT is controversial, however. As noted in the introduction, restoring macrophage function in a mouse RTT model was reported to alleviate some symptoms in one study, but not another [27, 28]. In addition, Schafer et al. suggested recently that while microglia may contribute to the RTT phenotype by enhancing engulfment and the elimination of presynaptic inputs at the end stage of disease, specific loss or gain of *Mecp2* expression in microglia were not primary events [29]. On the other hand, *Mecp2* deficiency leads to dysregulation of inflammatory responses in microglia and macrophages, which could alter their function and contribute to some aspects of disease pathogenesis [56], which is supported by our findings .

With these findings in mind, we carried out a transcriptome study in microglia derived from female Het mice, a model that more closely mimics clinical RTT, which occurs in females. One of our significant findings was that genes coding for nine HSP proteins were markedly decreased in microglia from pre-phenotypic female RTT mice. By contrast, no differences in HSP gene expression were detected in the 24-week samples derived from mice that had developed neurological symptoms. This suggests that in pre-phenotypic female mice, microglia could be susceptible to hyperthermia and perhaps other cellular stressors that function through the HSP pathway, such as oxidative stress, hypoxic stress, and inflammation. HSPs are molecular chaperones that play a role in brain function by preventing protein misfolding and by promoting the degradation of proteins that are improperly folded [69, 70]. Upon exposure to cellular stress, an increase in expression occurs, which protects cells from apoptosis. Although there are no studies suggesting that HSPs play a role in RTT, there is some evidence pointing to other forms of cellular stress having an impact. For example, reduced Mecp2 expression leads to redox imbalance and oxidative stress [71–73]. In addition, increased expression of hypoxia-induced transcripts was found in *Mecp2*-deficient microglia and peritoneal macrophages [56].

Another significant finding in our study is that both 5- and 24-week DEGs were significantly enriched for genes that are associated with both the M1 and M2 activation states, suggesting that neuroinflammation is an active process in both pre- and post-phenotypic RTT mice. Curiously, however, disparate M1 and M2 gene sets were differentially expressed in pre-phenotypic and phenotypic microglia. This could be due, perhaps, to a temporal difference in microglial activation in RTT that are specific to microglia per se, or exogenous, due to interactions with other cell types in the brain; microglia can be activated through contact with neurons via the pruning of weak, ineffective synapses, and with activated astrocytes, oligodendrocytes, and endothelial cells [74–76].

Disparate findings in pre-phenotypic and phenotypic microglia were also observed by Cronk et al. who showed that compared with control males, microglia have significantly smaller soma in pre-phenotypic *Mecp2*-null mice, but larger soma (indicative of microglial activation) in phenotypic mice [56].

Although overall, there was overlap with the Cronk et al. transcriptome findings on a general level (i.e., altered expression of inflammatory and cellular stress genes); there was limited overlap in DEGs per se, perhaps owing to differences in sex; we used females and their RNA-seq data were obtained using males, or temporal differences (early phenotype vs late phenotype).

Finally, the enrichment of DEGs that contribute to the ECM in the 5-week RTT samples is interesting in view of the contribution made by microglia, albeit modest compared with astrocytes and neurons, to ECM homeostasis [77–81]. Considering the importance of the ECM in maintaining synaptic function, the altered expression of ECM components and regulators in the pre-phenotypic phase could play some role in the synaptic dysfunction that ultimately leads to clinical symptoms.

Overall, our results suggest that the molecular alterations in *Mecp2*-deficient microglia are relatively small but certain genes may have important roles in modulating synaptic homeostasis and function.

A caveat to our findings is that the microglia from Het mice were not separated into WT and Mecp2-null cells. Thus, X chromosome skewing could have had an impact on our results. We computed the ratio of RNA-seq reads mapped to the deleted exons (exons 3 and 4) vs the reads mapped to the undeleted exons (exons 1 and 2) and then compared the ratios between Mecp2 Het and WT samples. Based on this, we estimate that the Mecp2-null cells were probably present in ~40% of the cells used for RNA sequencing (data not shown) suggesting that X chromosome skewing did not have a significant impact on our findings. However, follow-up single-cell sequencing studies will be necessary to better resolve this.

In addition, follow-up functional studies are needed to validate our findings, in particular, whether microglia derived from pre-phenotypic RTT mice show an altered response to heat shock and other stressors that require an intact HSP response, the dysfunction of which might compromise microglial function, contributing to the development of symptoms later in life.

## Conclusions

Transcriptome analysis was carried out on microglia from control female mice and heterozygous female mice carrying one Mecp2-null allele—a mouse model of Rett syndrome—at two time points; 5 weeks, prior to the onset of neurological symptoms (pre-phenotypic) and 24 weeks, after the development of neurological symptoms (phenotypic). Genes involved in innate immunity and in M1 and M2 macrophage activation were differentially expressed at both time points, although different sets of genes were found at the two time points. In addition, a number of heat shock genes were differentially expressed at 5 weeks, but not at 24 weeks. These findings suggest that pre-phenotypic female mice may have alterations in their capacity to response to heat stress and other stressors that function through the HSP pathway, as well as dysregulated expression of genes involved in innate immunity, both of which might contribute to the later development of neurological symptoms.

## Additional files

Additional file 1: Figure S1. Representative histograms from the FACS analysis CD11b+ and CD11b− fractions obtained in the microglial isolation procedure. (JPG 62 kb)

Additional file 2: Table S1. Total number of reads and mapped reads in 5- and 24-week Het females and WT controls. The mapping statistics were from tophat2 alignment report. QC of mapping results was done with RSeQC [82]. (XLSX 11 kb)

Additional file 3: Table S2. Entire gene list for Het vs WT in 5-week (sheet 1) and 24-week microglia (sheet 2). The genes are listing in descending order according to the adjusted *p* value (padj). Sheet 3 shows the number of DEGs in the 5-week samples after repeating the analysis five times by randomly sampling from the 5-week samples the numbers of RNA-seq reads matching to the read depth of the week 24 samples. (XLSX 7264 kb)

Additional file 4: Figure S2. Principal components analysis (PCA) was carried out on the top 5000 most variable genes as defined by the software DESeq2. The samples could be separated using PC1 and PC3. (JPG 47 kb)

Additional file 5: Figure S3. A heat map shows the expression level of all week 5 DEGs across all samples, where expression levels here are quantified by normalized read counts (log10-transformed). As seen in the heat map, similar levels of expression were found in the week 4 and week 24 samples. (JPG 103 kb)

Additional file 6: Table S3. Enrichment analysis of DEGs using the ToppGene suite. 5-week down-regulated DEGs (sheet 1); 5-week up-regulated DEGs (sheet 2); 24-week down-regulated DEGs (sheet 3); and 24-week up-regulated DEGs (sheet 4). (XLSX 7206 kb)

Additional file 7: Table S4. DEGs that overlap with M1- and M2-activated genes. Week 5 and week 24 DEGs were compared with genes activated in mouse macrophage following M1 and M2 activation found by Jablonski et al. as described in the Methods section [36]. Each sheet contains the overlap of up- or down-regulated genes with M1- or M2-activated genes. (XLSX 502 kb)

## Abbreviations

ASD: Autism spectrum disorders
C4: Complement component 4
DEG: Differentially expressed genes
ECM: Extracellular matrix
ES: Embryonic stem cells
GO: Gene ontogeny
Het: Heterozygote
HSP: Heat shock protein
MHC: Major histocompatibility
PCA: Principal component analysis
qPCR: Quantitative real-time PCR
RTT: Rett syndrome

## References

[1] Amir RE, Van den Veyver IB, Wan M, Tran CQ, Francke U, Zoghbi HY (1999). Rett syndrome is caused by mutations in X-linked MECP2, encoding methyl-CpG-binding protein 2. Nat Genet.

[2] Pohodich AE, Zoghbi HY (2015). Rett syndrome: disruption of epigenetic control of postnatal neurological functions. Hum Mol Genet.

[3] Mellen M, Ayata P, Dewell S, Kriaucionis S, Heintz N (2012). MeCP2 binds to 5hmC enriched within active genes and accessible chromatin in the nervous system. Cell.

[4] Guo JU, Su Y, Shin JH, Shin J, Li H, Xie B, Zhong C, Hu S, Le T, Fan G, Zhu H, Chang Q, Gao Y, Ming GL, Song H (2014). Distribution, recognition and regulation of non-CpG methylation in the adult mammalian brain. Nat Neurosci.

[5] Cheng TL, Wang Z, Liao Q, Zhu Y, Zhou WH, Xu W, Qiu Z (2014). MeCP2 suppresses nuclear microRNA processing and dendritic growth by regulating the DGCR8/Drosha complex. Dev Cell.

[6] Bianciardi L, Fichera M, Failla P, Di Marco C, Grozeva D, Mencarelli MA, Spiga O, Mari F, Meloni I, Raymond L, Renieri A, Romano C, Ariani F. MECP2 missense mutations outside the canonical MBD and TRD domains in males with intellectual disability. J Hum Genet. 2016;61(2):95–101. 10.1038/jhg.2015.118 PMC4770571 26490184

[7] Adegbola AA, Gonzales ML, Chess A, LaSalle JM, Cox GF (2009). A novel hypomorphic MECP2 point mutation is associated with a neuropsychiatric phenotype. Hum Genet.

[8] Hara M, Ohba C, Yamashita Y, Saitsu H, Matsumoto N, Matsuishi T (2015). De novo SHANK3 mutation causes Rett syndrome-like phenotype in a female patient. Am J Med Genet A.

[9] Sajan SA, Jhangiani SN, Muzny DM, Gibbs RA, Lupski JR, Glaze DG, Kaufmann WE, Skinner SA, Annese F, Friez MJ, Lane J, Percy AK, Neul JL. Enrichment of mutations in chromatin regulators in people with Rett syndrome lacking mutations in MECP2. Genet Med. 2017;19(1):13–9. 10.1038/gim.2016.42 PMC5107176 27171548

[10] Lucariello M, Vidal E, Vidal S, Saez M, Roa L, Huertas D, Pineda M, Dalfo E, Dopazo J, Jurado P, Armstrong J, Esteller M (2016). Whole exome sequencing of Rett syndrome-like patients reveals the mutational diversity of the clinical phenotype. Hum Genet.

[11] McCarthy SE, Gillis J, Kramer M, Lihm J, Yoon S, Berstein Y, Mistry M, Pavlidis P, Solomon R, Ghiban E, Antoniou E, Kelleher E, O'Brien C, Donohoe G, Gill M, Morris DW, McCombie WR, Corvin A (2014). De novo mutations in schizophrenia implicate chromatin remodeling and support a genetic overlap with autism and intellectual disability. Mol Psychiatry.

[12] Wong EH, So HC, Li M, Wang Q, Butler AW, Paul B, Wu HM, Hui TC, Choi SC, So MT, Garcia-Barcelo MM, McAlonan GM, Chen EY, Cheung EF, Chan RC, Purcell SM, Cherny SS, Chen RR, Li T, Sham PC (2014). Common variants on Xq28 conferring risk of schizophrenia in Han Chinese. Schizophr Bull.

[13] Lombardi LM, Baker SA, Zoghbi HY (2015). MECP2 disorders: from the clinic to mice and back. J Clin Invest.

[14] Chen RZ, Akbarian S, Tudor M, Jaenisch R (2001). Deficiency of methyl-CpG binding protein-2 in CNS neurons results in a Rett-like phenotype in mice. Nat Genet.

[15] He LJ, Liu N, Cheng TL, Chen XJ, Li YD, Shu YS, Qiu ZL, Zhang XH (2014). Conditional deletion of Mecp2 in parvalbumin-expressing GABAergic cells results in the absence of critical period plasticity. Nat Commun.

[16] Giacometti E, Luikenhuis S, Beard C, Jaenisch R (2007). Partial rescue of MeCP2 deficiency by postnatal activation of MeCP2. Proc Natl Acad Sci U S A.

[17] Xu X, Miller EC, Pozzo-Miller L (2014). Dendritic spine dysgenesis in Rett syndrome. Front Neuroanat.

[18] Jentarra GM, Olfers SL, Rice SG, Srivastava N, Homanics GE, Blue M, Naidu S, Narayanan V (2010). Abnormalities of cell packing density and dendritic complexity in the MeCP2 A140V mouse model of Rett syndrome/X-linked mental retardation. BMC Neurosci.

[19] Ure K, Lu H, Wang W, Ito-Ishida A, Wu Z, He LJ, Sztainberg Y, Chen W, Tang J, Zoghbi HY. Restoration of Mecp2 expression in GABAergic neurons is sufficient to rescue multiple disease features in a mouse model of Rett syndrome. Elife. 2016;5:10.7554/eLife.14198. 10.7554/eLife.14198 PMC4946897 27328321

[20] Meng X, Wang W, Lu H, He LJ, Chen W, Chao ES, Fiorotto ML, Tang B, Herrera JA, Seymour ML, Neul JL, Pereira FA, Tang J, Xue M, Zoghbi HY. Manipulations of MeCP2 in glutamatergic neurons highlight their contributions to Rett and other neurological disorders. Elife. 2016;5:10.7554/eLife.14199. 10.7554/eLife.14199 PMC4946906 27328325

[21] Weng SM, McLeod F, Bailey ME, Cobb SR (2011). Synaptic plasticity deficits in an experimental model of Rett syndrome: long-term potentiation saturation and its pharmacological reversal. Neuroscience.

[22] Ramocki MB, Tavyev YJ, Peters SU (2010). The MECP2 duplication syndrome. Am J Med Genet A.

[23] Nageshappa S, Carromeu C, Trujillo CA, Mesci P, Espuny-Camacho I, Pasciuto E, Vanderhaeghen P, Verfaillie CM, Raitano S, Kumar A, Carvalho CM, Bagni C, Ramocki MB, Araujo BH, Torres LB, Lupski JR, Van Esch H, Muotri AR. Altered neuronal network and rescue in a human MECP2 duplication model. Mol Psychiatry. 2016;21(2):178–88. 10.1038/mp.2015.128 PMC4720528 26347316

[24] Lioy DT, Garg SK, Monaghan CE, Raber J, Foust KD, Kaspar BK, Hirrlinger PG, Kirchhoff F, Bissonnette JM, Ballas N, Mandel G (2011). A role for glia in the progression of Rett’s syndrome. Nature.

[25] Nguyen MV, Felice CA, Du F, Covey MV, Robinson JK, Mandel G, Ballas N (2013). Oligodendrocyte lineage cells contribute unique features to Rett syndrome neuropathology. J Neurosci.

[26] Jin LW, Horiuchi M, Wulff H, Liu XB, Cortopassi GA, Erickson JD, Maezawa I (2015). Dysregulation of glutamine transporter SNAT1 in Rett syndrome microglia: a mechanism for mitochondrial dysfunction and neurotoxicity. J Neurosci.

[27] Derecki NC, Cronk JC, Lu Z, Xu E, Abbott SB, Guyenet PG, Kipnis J (2012). Wild-type microglia arrest pathology in a mouse model of Rett syndrome. Nature.

[28] Wang J, Wegener JE, Huang TW, Sripathy S, De Jesus-Cortes H, Xu P, Tran S, Knobbe W, Leko V, Britt J, Starwalt R, McDaniel L, Ward CS, Parra D, Newcomb B, Lao U, Nourigat C, Flowers DA, Cullen S, Jorstad NL, Yang Y, Glaskova L, Vingeau S, Kozlitina J, Yetman MJ, Jankowsky JL, Reichardt SD, Reichardt HM, Gartner J, Bartolomei MS, Fang M, Loeb K, Keene CD, Bernstein I, Goodell M, Brat DJ, Huppke P, Neul JL, Bedalov A, Pieper AA (2015). Wild-type microglia do not reverse pathology in mouse models of Rett syndrome. Nature.

[29] Schafer DP, Heller CT, Gunner G, Heller M, Gordon C, Hammond T, Wolf Y, Jung S, Stevens B. Microglia contribute to circuit defects in Mecp2 null mice independent of microglia-specific loss of Mecp2 expression. Elife. 2016; 5:10.7554/eLife.15224. 10.7554/eLife.15224 PMC4961457 27458802

[30] Guy J, Hendrich B, Holmes M, Martin JE, Bird A (2001). A mouse Mecp2-null mutation causes neurological symptoms that mimic Rett syndrome. Nat Genet.

[31] Lieu CA, Chinta SJ, Rane A, Andersen JK (2013). Age-related behavioral phenotype of an astrocytic monoamine oxidase-B transgenic mouse model of Parkinson’s disease. PLoS One.

[32] Kim D, Pertea G, Trapnell C, Pimentel H, Kelley R, Salzberg SL (2013). TopHat2: accurate alignment of transcriptomes in the presence of insertions, deletions and gene fusions. Genome Biol.

[33] Anders S, Pyl PT, Huber W (2015). HTSeq—a Python framework to work with high-throughput sequencing data. Bioinformatics.

[34] Love MI, Huber W, Anders S (2014). Moderated estimation of fold change and dispersion for RNA-seq data with DESeq2. Genome Biol.

[35] Chen J, Bardes EE, Aronow BJ, Jegga AG (2009). ToppGene Suite for gene list enrichment analysis and candidate gene prioritization. Nucleic Acids Res.

[36] Jablonski KA, Amici SA, Webb LM, Ruiz-Rosado Jde D, Popovich PG, Partida-Sanchez S, Guerau-de-Arellano M (2015). Novel markers to delineate murine M1 and M2 macrophages. PLoS One.

[37] Smyth GK, Michaud J, Scott HS (2005). Use of within-array replicate spots for assessing differential expression in microarray experiments. Bioinformatics.

[38] Hochberg Y, Benjamini Y (1990). More powerful procedures for multiple significance testing. Stat Med.

[39] Wang P, Lin M, Pedrosa E, Hrabovsky A, Zhang Z, Guo W, Lachman HM, Zheng D (2015). CRISPR/Cas9-mediated heterozygous knockout of the autism gene CHD8 and characterization of its transcriptional networks in neurodevelopment. Mol Autism.

[40] Lin M, Pedrosa E, Hrabovsky A, Chen J, Puliafito BR, Gilbert SR, Zheng D, Lachman HM (2016). Integrative transcriptome network analysis of iPSC-derived neurons from schizophrenia and schizoaffective disorder patients with 22q11.2 deletion. BMC Syst Biol.

[41] Della Ragione F, Vacca M, Fioriniello S, Pepe G, D'Esposito M. MECP2, a multi-talented modulator of chromatin architecture. Brief Funct Genomics. 2016;15(6):420–31. 10.1093/bfgp/elw023 27296483

[42] Ansari MA (2015). Temporal profile of M1 and M2 responses in the hippocampus following early 24 h of neurotrauma. J Neurol Sci.

[43] Reus GZ, Fries GR, Stertz L, Badawy M, Passos IC, Barichello T, Kapczinski F, Quevedo J (2015). The role of inflammation and microglial activation in the pathophysiology of psychiatric disorders. Neuroscience.

[44] Onore CE, Schwartzer JJ, Careaga M, Berman RF, Ashwood P (2014). Maternal immune activation leads to activated inflammatory macrophages in offspring. Brain Behav Immun.

[45] Gupta S, Ellis SE, Ashar FN, Moes A, Bader JS, Zhan J, West AB, Arking DE (2014). Transcriptome analysis reveals dysregulation of innate immune response genes and neuronal activity-dependent genes in autism. Nat Commun.

[46] Nowaczyk MJ, Huang L, Tarnopolsky M, Schwartzentruber J, Majewski J, Bulman DE, FORGE Canada Consortium, Care4Rare Canada Consortium, Hartley T, Boycott KM. A novel multisystem disease associated with recessive mutations in the tyrosyl-tRNA synthetase (YARS) gene. Am J Med Genet A. 2016. 10.1002/ajmg.a.37973 27633801

[47] Fensterl V, Sen GC (2015). Interferon-induced Ifit proteins: their role in viral pathogenesis. J Virol.

[48] Dourlen P, Fernandez-Gomez FJ, Dupont C, Grenier-Boley B, Bellenguez C, Obriot H, Caillierez R, Sottejeau Y, Chapuis J, Bretteville A, Abdelfettah F, Delay C, Malmanche N, Soininen H, Hiltunen M, Galas MC, Amouyel P, Sergeant N, Buee L, Lambert JC, Dermaut B. Functional screening of Alzheimer risk loci identifies PTK2B as an in vivo modulator and early marker of Tau pathology. Mol Psychiatry. 2016. doi:10.1038/mp.2016.59. [Epub ahead of print] 10.1038/mp.2016.59 PMC5444024 27113998

[49] Beecham GW, Hamilton K, Naj AC, Martin ER, Huentelman M, Myers AJ, Corneveaux JJ, Hardy J, Vonsattel JP, Younkin SG, Bennett DA, De Jager PL, Larson EB, Crane PK, Kamboh MI, Kofler JK, Mash DC, Duque L, Gilbert JR, Gwirtsman H, Buxbaum JD, Kramer P, Dickson DW, Farrer LA, Frosch MP, Ghetti B, Haines JL, Hyman BT, Kukull WA, Mayeux RP, Pericak-Vance MA, Schneider JA, Trojanowski JQ, Reiman EM, Schellenberg GD, Montine TJ, Alzheimer’s Disease Genetics Consortium (ADGC) (2014). Genome-wide association meta-analysis of neuropathologic features of Alzheimer's disease and related dementias. PLoS Genet.

[50] Schott J, Reitter S, Philipp J, Haneke K, Schafer H, Stoecklin G (2014). Translational regulation of specific mRNAs controls feedback inhibition and survival during macrophage activation. PLoS Genet.

[51] Lit L, Sharp FR, Bertoglio K, Stamova B, Ander BP, Sossong AD, Hendren RL (2012). Gene expression in blood is associated with risperidone response in children with autism spectrum disorders. Pharmacogenomics J.

[52] Hossain M, Qadri SM, Xu N, Su Y, Cayabyab FS, Heit B, Liu L (2015). Endothelial LSP1 modulates extravascular neutrophil chemotaxis by regulating nonhematopoietic vascular PECAM-1 expression. J Immunol.

[53] Ma Y, Bao J, Zhao X, Shen H, Lv J, Ma S, Zhang X, Li Z, Wang S, Wang Q, Ji J (2013). Activated cyclin-dependent kinase 5 promotes microglial phagocytosis of fibrillar beta-amyloid by up-regulating lipoprotein lipase expression. Mol Cell Proteomics.

[54] Gong H, Dong W, Rostad SW, Marcovina SM, Albers JJ, Brunzell JD, Vuletic S (2013). Lipoprotein lipase (LPL) is associated with neurite pathology and its levels are markedly reduced in the dentate gyrus of Alzheimer’s disease brains. J Histochem Cytochem.

[55] Derecki NC, Cronk JC, Kipnis J (2013). The role of microglia in brain maintenance: implications for Rett syndrome. Trends Immunol.

[56] Cronk JC, Derecki NC, Ji E, Xu Y, Lampano AE, Smirnov I, Baker W, Norris GT, Marin I, Coddington N, Wolf Y, Turner SD, Aderem A, Klibanov AL, Harris TH, Jung S, Litvak V, Kipnis J (2015). Methyl-CpG binding protein 2 regulates microglia and macrophage gene expression in response to inflammatory stimuli. Immunity.

[57] Voineagu I, Eapen V (2013). Converging pathways in autism spectrum disorders: interplay between synaptic dysfunction and immune responses. Front Hum Neurosci.

[58] Voineagu I, Wang X, Johnston P, Lowe JK, Tian Y, Horvath S, Mill J, Cantor RM, Blencowe BJ, Geschwind DH (2011). Transcriptomic analysis of autistic brain reveals convergent molecular pathology. Nature.

[59] Schafer DP, Lehrman EK, Kautzman AG, Koyama R, Mardinly AR, Yamasaki R, Ransohoff RM, Greenberg ME, Barres BA, Stevens B (2012). Microglia sculpt postnatal neural circuits in an activity and complement-dependent manner. Neuron.

[60] Paolicelli RC, Bolasco G, Pagani F, Maggi L, Scianni M, Panzanelli P, Giustetto M, Ferreira TA, Guiducci E, Dumas L, Ragozzino D, Gross CT (2011). Synaptic pruning by microglia is necessary for normal brain development. Science.

[61] Grayson DR, Guidotti A (2016). Merging data from genetic and epigenetic approaches to better understand autistic spectrum disorder. Epigenomics.

[62] Sekar A, Bialas AR, de Rivera H, Davis A, Hammond TR, Kamitaki N, Tooley K, Presumey J, Baum M, Van Doren V, Genovese G, Rose SA, Handsaker RE, Daly MJ, Carroll MC, Stevens B, McCarroll SA, Schizophrenia Working Group of the Psychiatric Genomics Consortium (2016). Schizophrenia risk from complex variation of complement component 4. Nature.

[63] Havik B, Le Hellard S, Rietschel M, Lybaek H, Djurovic S, Mattheisen M, Muhleisen TW, Degenhardt F, Priebe L, Maier W, Breuer R, Schulze TG, Agartz I, Melle I, Hansen T, Bramham CR, Nothen MM, Stevens B, Werge T, Andreassen OA, Cichon S, Steen VM (2011). The complement control-related genes CSMD1 and CSMD2 associate to schizophrenia. Biol Psychiatry.

[64] Cukier HN, Dueker ND, Slifer SH, Lee JM, Whitehead PL, Lalanne E, Leyva N, Konidari I, Gentry RC, Hulme WF, Booven DV, Mayo V, Hofmann NK, Schmidt MA, Martin ER, Haines JL, Cuccaro ML, Gilbert JR, Pericak-Vance MA (2014). Exome sequencing of extended families with autism reveals genes shared across neurodevelopmental and neuropsychiatric disorders. Mol Autism.

[65] Zhang Y, Chen K, Sloan SA, Bennett ML, Scholze AR, O'Keeffe S, Phatnani HP, Guarnieri P, Caneda C, Ruderisch N, Deng S, Liddelow SA, Zhang C, Daneman R, Maniatis T, Barres BA, Wu JQ (2014). An RNA-sequencing transcriptome and splicing database of glia, neurons, and vascular cells of the cerebral cortex. J Neurosci.

[66] Correia CT, Conceicao IC, Oliveira B, Coelho J, Sousa I, Sequeira AF, Almeida J, Cafe C, Duque F, Mouga S, Roberts W, Gao K, Lowe JK, Thiruvahindrapuram B, Walker S, Marshall CR, Pinto D, Nurnberger JI, Scherer SW, Geschwind DH, Oliveira G, Vicente AM (2014). Recurrent duplications of the annexin A1 gene (ANXA1) in autism spectrum disorders. Mol Autism.

[67] Costain G, Lionel AC, Fu F, Stavropoulos DJ, Gazzellone MJ, Marshall CR, Scherer SW, Bassett AS (2014). Adult neuropsychiatric expression and familial segregation of 2q13 duplications. Am J Med Genet B Neuropsychiatr Genet.

[68] He Z, O'Roak BJ, Smith JD, Wang G, Hooker S, Santos-Cortez RL, Li B, Kan M, Krumm N, Nickerson DA, Shendure J, Eichler EE, Leal SM (2014). Rare-variant extensions of the transmission disequilibrium test: application to autism exome sequence data. Am J Hum Genet.

[69] De Maio A (2014). Extracellular Hsp70: export and function. Curr Protein Pept Sci.

[70] Amor S, Bugiani M, van Noort JM. Heat shock proteins: old and novel roles in neurodegenerative diseases in the central nervous system. CNS Neurol Disord Drug Targets. 2016. [Epub ahead of print] 10.2174/1871527315666161031125317 27804858

[71] Pecorelli A, Cervellati C, Hayek J, Valacchi G. OxInflammation in Rett syndrome. Int J Biochem Cell Biol. 2016;81(Pt B):246–53. 10.1016/j.biocel.2016.07.015 27425398

[72] Filosa S, Pecorelli A, D'Esposito M, Valacchi G, Hajek J (2015). Exploring the possible link between MeCP2 and oxidative stress in Rett syndrome. Free Radic Biol Med.

[73] Signorini C, De Felice C, Leoncini S, Moller RS, Zollo G, Buoni S, Cortelazzo A, Guerranti R, Durand T, Ciccoli L, D'Esposito M, Ravn K, Hayek J (2016). MECP2 duplication syndrome: evidence of enhanced oxidative stress. A comparison with Rett syndrome. PLoS One.

[74] Domingues HS, Portugal CC, Socodato R, Relvas JB (2016). Oligodendrocyte, astrocyte, and microglia crosstalk in myelin development, damage, and repair. Front Cell Dev Biol.

[75] Luo X, Tai WL, Sun L, Pan Z, Xia Z, Chung SK, Cheung CW. Crosstalk between astrocytic CXCL12 and microglial CXCR4 contributes to the development of neuropathic pain. Mol Pain. 2016;12:10.1177/1744806916636385. Print 2016. 10.1177/1744806916636385 PMC4956184 27030717

[76] da Fonseca AC, Matias D, Garcia C, Amaral R, Geraldo LH, Freitas C, Lima FR (2014). The impact of microglial activation on blood-brain barrier in brain diseases. Front Cell Neurosci.

[77] Blank T, Prinz M (2013). Microglia as modulators of cognition and neuropsychiatric disorders. Glia.

[78] del Zoppo GJ, Frankowski H, Gu YH, Osada T, Kanazawa M, Milner R, Wang X, Hosomi N, Mabuchi T, Koziol JA (2012). Microglial cell activation is a source of metalloproteinase generation during hemorrhagic transformation. J Cereb Blood Flow Metab.

[79] Nuttall RK, Silva C, Hader W, Bar-Or A, Patel KD, Edwards DR, Yong VW (2007). Metalloproteinases are enriched in microglia compared with leukocytes and they regulate cytokine levels in activated microglia. Glia.

[80] Dzyubenko E, Gottschling C, Faissner A (2016). Neuron-glia interactions in neural plasticity: contributions of neural extracellular matrix and perineuronal nets. Neural Plast.

[81] Kelly EA, Russo AS, Jackson CD, Lamantia CE, Majewska AK (2015). Proteolytic regulation of synaptic plasticity in the mouse primary visual cortex: analysis of matrix metalloproteinase 9 deficient mice. Front Cell Neurosci.

[82] Wang L, Wang S, Li W (2012). RSeQC: quality control of RNA-seq experiments. Bioinformatics.

