# Supplementary figures and images for: Transcriptome analysis of microglia in a mouse model of Rett syndrome: differential expression of genes associated with microglia/macrophage activation and cellular stress
Source: Mol Autism. 2017 Mar 29;8:17. doi: 10.1186/s13229-017-0134-z (PMC5372344; doi:10.1186/s13229-017-0134-z)

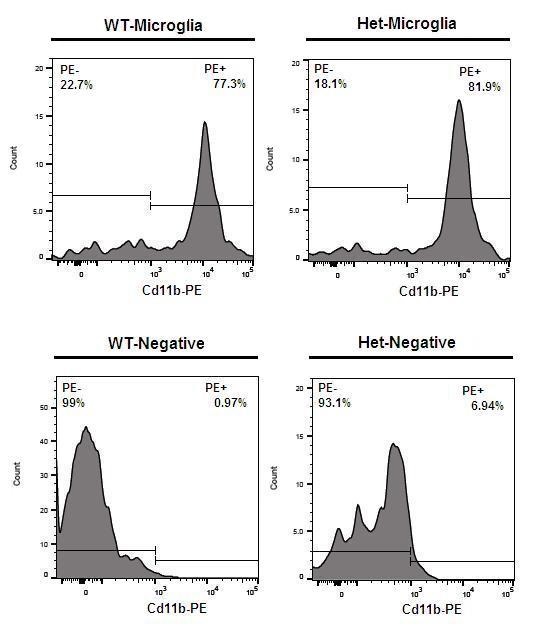

Supplement: Supplementary file 1 — Representative histograms from the FACS analysis CD11b+ and CD11b− fractions obtained in the microglial isolation procedure. (JPG 62 kb) [file 13229_2017_134_MOESM1_ESM.jpg]

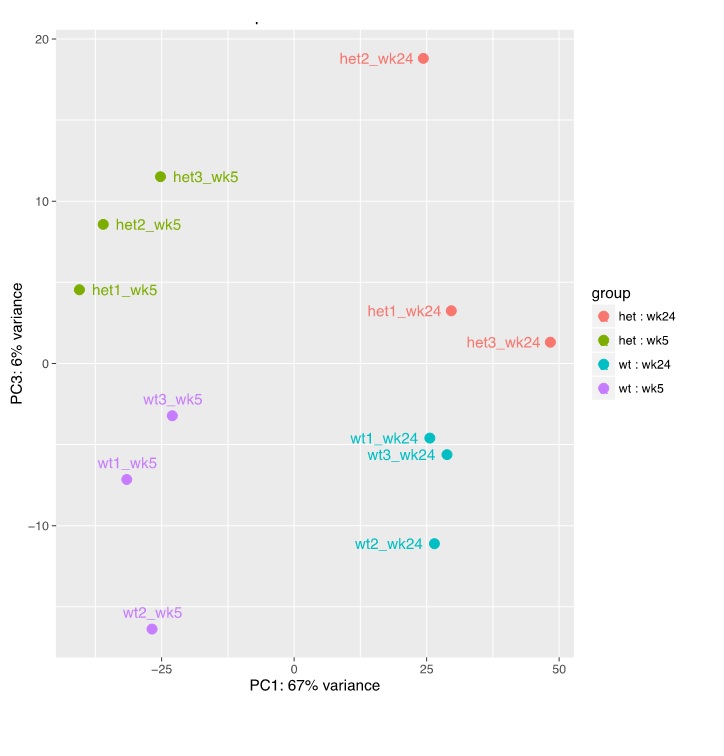

Supplement: Supplementary file 4 — Principal components analysis (PCA) was carried out on the top 5000 most variable genes as defined by the software DESeq2. The samples could be separated using PC1 and PC3. (JPG 47 kb) [file 13229_2017_134_MOESM4_ESM.jpg]

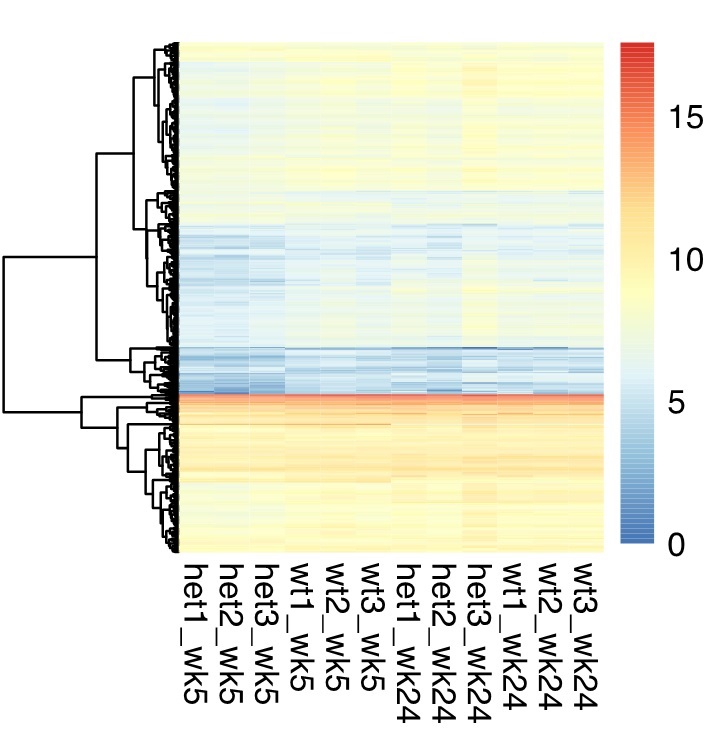

Supplement: Supplementary file 5 — A heat map shows the expression level of all week 5 DEGs across all samples, where expression levels here are quantified by normalized read counts (log10-transformed). As seen in the heat map, similar levels of expression were found in the week 4 and week 24 samples. (JPG 103 kb) [file 13229_2017_134_MOESM5_ESM.jpg]
